# Sentinel Node Navigation Surgery in Gastric Cancer: A Systematic Review of Diagnostic Test Accuracy and Clinical Outcomes

**DOI:** 10.3390/cancers18203332

**Published:** 2026-10-15

**Authors:** Jonathan W. Kim, Helen D. Kim, Elliott Lee, Steve Kwon

**Affiliations:** 1 College of Arts and Sciences, University of Pennsylvania, Philadelphia, PA 19104, USA; 2 The Warren Alpert Medical School, Brown University, Providence, RI 02912, USA; helen_d_kim@brown.edu; 3 Roger Williams Surgery and Cancer Outcomes Research and Equity (RWSCORE) Center, Roger Williams Medical Center, Providence, RI 02908, USA; 4 Department of Surgery, Division of Surgical Oncology, School of Medicine, Yale University, 330 Cedar Street FMB 130E, New Haven, CT 06510, USA

**Keywords:** early gastric cancer, sentinel node navigation surgery, sentinel lymph node mapping

## Abstract

Background/Objectives: Gastric cancer remains a leading cause of cancer mortality. However, earlier detection has increased the number of cases suitable for organ-preserving treatment. Sentinel node navigation surgery has been proposed as an alternative to standard gastrectomy for early gastric cancer. The safety of this procedure hinges on how accurately sentinel node mapping predicts nodal status. This systematic review synthesizes its diagnostic accuracy and outcome data. Methods: We searched PubMed/MEDLINE, Embase, CENTRAL, the Web of Science Core Collection, and Scopus from inception without date limits, along with two trial registries; screening was restricted to English-language reports. Estimates and exact binomial (Clopper–Pearson) 95% confidence intervals were recalculated from each study’s reported contingency counts using a consistent statistical method. Each included cohort was appraised using QUADAS-2. Results: Eight cohorts contributed per-patient accuracy data, and three contributed per-node molecular comparisons. Detection rates were 97.1% or higher in every cohort entry, with the exception of the naked-eye dye arm of a within-cohort comparison and the smallest feasibility series. Sensitivity was 93.0% (53/57; 95% CI, 83.0–98.1) in the single adequately powered treatment-naive cohort, compared to 69.6% (16/23; 47.1–86.8) in the single prospective trial conducted after non-curative endoscopic resection. Sensitivity also depended on the thoroughness of sentinel node examination: within one cohort, the same patients yielded a sensitivity of 53.6% with single-plane frozen section versus 85.7% with multiple paraffin sections. Therefore, estimates were not pooled. No appraised cohort demonstrated a low risk of bias across all four QUADAS-2 domains. Conclusions: Stomach preservation guided by sentinel node mapping may be a reasonable approach for treatment-naive disease in experienced centers. However, it is not supported after non-curative endoscopic resection.

## Simple Summary

Standard surgery for early gastric cancer involves removing a large part of the stomach and nearby lymph nodes. This extensive removal occurs even though most early tumors have not yet spread to these nodes. Since nodal status cannot be confirmed before surgery, many patients undergo unnecessary tissue removal, often leading to long-term difficulties with eating, weight management, and nutrition. Sentinel node navigation surgery offers an alternative. A tracer is injected around the tumor to identify the first draining lymph nodes. If these “sentinel” nodes are found to be clear of cancer during the operation, the rest of the stomach and its associated nodes are left intact. This review re-evaluated the accuracy reported in published studies. The mapping technique appears accurate enough for consideration in newly diagnosed, untreated patients. However, it is not recommended for patients who have already undergone endoscopic tumor removal. The accuracy of this technique also depends on the thoroughness with which the removed nodes are examined under a microscope, not solely on how they are identified during surgery.

## 1. Introduction

Gastric cancer is a leading cause of cancer-related death worldwide, with the stage at diagnosis varying widely by region [1]. In Japan and Korea, national screening programs have led to early detection in over half of cases, compared to roughly one in five in most Western countries [1]. Consequently, surgeons in screening regions frequently encounter patients with tumors confined to the mucosa or submucosa, which offer a high probability of cure [2]. For these patients, long-term well-being is more influenced by the functional impact of surgery than by oncological control [3,4].

Currently, gastrectomy with D1+ or D2 lymphadenectomy remains the standard treatment, partly because nodal status cannot be determined preoperatively [5]. While resection offers high survival rates (the Japanese nationwide registry reports a five-year survival of 91.5% for pathological stage IA disease [2]), the functional costs are substantial. Patients typically lose about 6% of their body weight after distal gastrectomy and 15% after total gastrectomy within a year. Micronutrient deficiency, dumping syndrome, bile reflux, and reduced bone density are common, necessitating dedicated follow-up [3,4].

The extent of nodal dissection further exacerbates these burdens. Clearing lymph nodes beyond the perigastric region requires the division of the celiac and hepatic branches of the vagus nerve. A randomized trial in early gastric cancer demonstrated that preserving these branches reduced diarrhea and appetite loss at 12 months [6], a finding supported by a subsequent pooled analysis [7].

Despite these considerations, the prior probability of nodal disease is low, affecting approximately 3% of mucosal and 20% of submucosal tumors [8]. This means that most patients undergoing radical gastrectomy for early gastric cancer do not benefit from extensive lymphadenectomy or the associated resection [5,8].

Endoscopic submucosal dissection (ESD) offers a solution for some cases. Lesions meeting the eCura A or B criteria carry a very low risk of nodal involvement and allow the stomach to remain intact [5]. However, tumors outside these curative criteria, or those found to have been non-curatively resected on final pathology, present a dilemma. Their nodal risk is low but not negligible [5,8,9]. Current guidelines recommend gastrectomy for these cases, yet most resected specimens ultimately prove node-negative [5,9].

Sentinel node navigation surgery (SNNS) was developed to identify which patients with early gastric cancer can be spared regional lymphadenectomy [10,11]. Whether it extends to the post-endoscopic-resection group above, where submucosal scarring may have redirected lymphatic drainage, is a separate question that this review addresses. This technique maps lymphatic drainage during surgery, examines the sentinel nodes, and, if these are clear, preserves the remaining regional nodes and most of the stomach [12,13]. Due to the multidirectional nature of gastric lymphatic drainage, the technique has evolved to dissect and assess the entire lymphatic basin containing the mapped nodes as a single unit [14]. The safety of SNNS relies on successful mapping in nearly every patient and on a tumor-free basin accurately predicting a tumor-free regional field [12,14].

This systematic review synthesizes the evidence for sentinel node navigation surgery in gastric cancer, with a particular focus on its diagnostic performance. Estimates were recalculated from reported contingency counts and are presented with exact binomial confidence intervals to allow direct comparison of precision across studies of varying sizes. Each included cohort was appraised using QUADAS-2 [15].

## 2. Materials and Methods

### 2.1. Protocol and Registration

This systematic review of diagnostic test accuracy adhered to the PRISMA 2020 statement [16] and its PRISMA-DTA extension [17]; the completed PRISMA 2020 and PRISMA-DTA checklists are provided as Table S4 and Table S5, respectively. The protocol was retrospectively registered with OSF Registries on 22 August 2026 (https://osf.io/ca7p2; PRISMA item 24a). All eligibility criteria, primary outcomes, and planned analyses were followed. The eligibility criteria and primary outcomes were finalized a priori, before the searches were run; each database was last searched on the date given in Supplementary Table S1 (between 10 and 19 August 2026), after which title/abstract and full-text screening of the retrieved records were completed, and the review was registered retrospectively on 22 August 2026. During data synthesis, a qualitative synthesis was chosen over pooled estimates (see Section 2.8).

### 2.2. Eligibility Criteria

Eligibility criteria were defined based on two components: diagnostic accuracy and outcomes. These criteria considered the population, index test, reference standard, target condition, and study design.

For the diagnostic accuracy component, eligible studies included adults with histologically confirmed gastric adenocarcinoma who underwent sentinel node mapping using any tracer strategy. This included patients who were treatment-naive, those with clinically early disease or those who had undergone non-curative endoscopic resection. Sentinel node status had to be determined histopathologically or molecularly. The reference standard was histopathology of regional nodes resected at lymphadenectomy for per-patient analyses, or of the paired node half for per-node analyses, with regional nodal metastasis as the target condition. We included randomized trials, cohort studies, and diagnostic accuracy designs if they reported, or allowed unambiguous reconstruction of, primary 2 × 2 contingency counts.

For the outcome component, we included randomized trials that compared stomach-preserving sentinel node navigation surgery with standard gastrectomy for oncological, surgical, or patient-reported outcomes, regardless of diagnostic accuracy reporting. These studies evaluate surgical strategy rather than test performance, so they are reported separately (Section 3.9) and were excluded from QUADAS-2 appraisal.

We excluded case reports, editorials, narrative reviews, animal and ex vivo studies, non-English publications, and conference abstracts lacking reconstructible outcome data. When multiple publications described the same patient cohort, all were retrieved, but the cohort was evaluated as a single study.

### 2.3. Information Sources and Search Strategy

**Figure 1:**
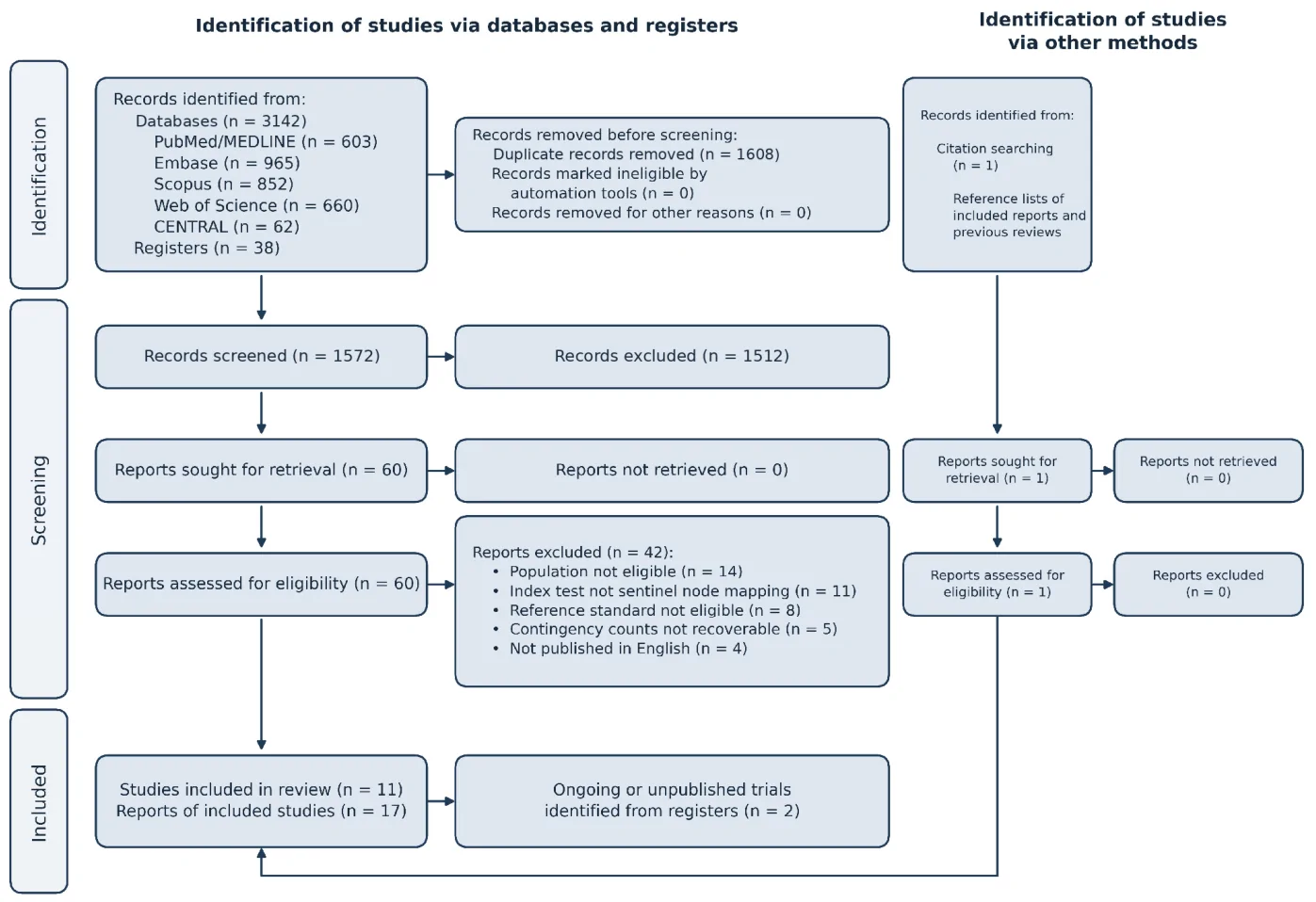
PRISMA flow diagram of study selection. Seventeen reports corresponding to 11 unique studies were included, along with 2 ongoing trial records. Multiple reports describing the same cohort were linked and counted as a single study. Thirteen reports contributed to the diagnostic accuracy stream and 4 to the outcome component; no report was counted twice, although the SENORITA phase III trial contributed to both. The full legend is given in the Supplementary Note S1.

We searched PubMed/MEDLINE, Embase, the Cochrane Central Register of Controlled Trials (CENTRAL), the Web of Science Core Collection, and Scopus from inception through the dates specified for each database in Supplementary Table S1. There were no language or publication-date restrictions during the initial search; however, non-English records were excluded during full-text screening (Figure 1). We hand-searched reference lists of included studies and reviews and screened trial registries (ClinicalTrials.gov, UMIN-CTR) for ongoing or unpublished trials. One additional report was identified through citation searching.

### 2.4. Study Selection

All retrieved records were exported to Zotero (https://www.zotero.org, accessed on 11 October 2026) for deduplication, with software-identified duplicates manually verified before removal. Two of three reviewers (J.W.K., H.D.K., and E.L.) independently screened titles, abstracts, and full texts in duplicate. Discrepancies were resolved by consensus or senior adjudication (S.K.). Records disputed at the title and abstract stage advanced to full-text review. Exclusion criteria were applied in a fixed sequence, stopping at the first unmet criterion, and a single primary reason was recorded for each excluded report (Figure 1).

### 2.5. Data Extraction

Data were extracted into a piloted spreadsheet, capturing study design, setting, population, sample size, tracer strategy, index test, reference standard, node-specific pathology protocols, detection rate, and primary 2 × 2 contingency counts. We recorded whether counts were directly reported or reconstructed. True-negative counts were derived from the number of patients with detected sentinel nodes and total node-positive cases. Values requiring reconstruction beyond direct subtractions are flagged in table footnotes.

Extraction was performed independently and in duplicate by J.W.K. and H.D.K. Discrepancies were resolved by checking source documents, performing arithmetic checks, or through adjudication by S.K.

### 2.6. Statistical Analysis

Detection rate, sensitivity, negative predictive value, and per-node molecular estimates were recalculated directly from reported counts. Exact binomial (Clopper–Pearson) 95% confidence intervals were computed in R (version 4.3.1; R Foundation for Statistical Computing, Vienna, Austria) using the stats package. Outcome and comparative statistics from the SENORITA phase III trial are reproduced as published. Minor numerical differences from source reports reflect the use of the exact binomial method rather than differences in the underlying data.

Specificity and overall accuracy are not reported for the per-patient analyses because full regional lymphadenectomy served as the reference standard. This precluded false positives and fixed specificity at 100%. The JCOG0302 study is the sole exception, as its index test was intraoperative frozen section and its reference standard was final paraffin. Specificity is reported for the per-node molecular comparisons (Section 3.8), where the paired node half provides an independent reference standard.

Data were evaluated at two levels: per-patient, testing whether sentinel node status predicts regional nodal status, and per-node, comparing molecular assays against paired histopathology. Sensitivity denominators follow each source report. When a cohort was evaluated under multiple index tests or pathology protocols, its results appear as linked rows under the study name to prevent double-counting. The two SENORITA trials are distinguished throughout: the phase III randomized trial in treatment-naive disease and the phase II SENORITA 2 trial after non-curative endoscopic resection.

### 2.7. Risk of Bias and Applicability

Risk of bias and applicability were evaluated across four domains using QUADAS-2 [15], with signaling questions tailored to the review question beforehand. Because sentinel node status in most studies was determined by the same paraffin examination serving as the reference standard, independent interpretation was judged partial incorporation rather than blinding and recorded as a concern in both the index test and reference standard domains. Reference standards were judged prone to misclassification where node examination was confined to a single plane or where non-uniform lymphadenectomy left metastatic nodes outside the resected field unexamined. QUADAS-2 and RoB 2 appraisals were performed independently by two reviewers (J.W.K. and H.D.K.); disagreements were resolved by discussion, with adjudication by a third reviewer (S.K.) where consensus could not be reached. Each domain was rated as having low or some concerns or a high risk of bias, with a low rating requiring affirmative answers across all signaling questions. We use the label “some concerns” (as in RoB 2) rather than the standard QUADAS-2 “unclear” category for the intermediate rating, because these ratings denote identified but non-critical limitations—chiefly partial incorporation of the index test into the reference standard—rather than an absence of information.

Risk of bias was assessed separately after extraction to keep the record of what each study did distinct from the judgment of how well it was done. Linked rows representing single cohorts under altered protocols were appraised separately to directly evaluate the protocol comparison. The outcome-stream randomized trial was evaluated using the Cochrane RoB 2 tool [18] (Section 3.9).

### 2.8. Synthesis

Diagnostic estimates were synthesized qualitatively rather than pooled via meta-analysis. Pooling was precluded by structural heterogeneity in how index tests and reference standards were paired across studies, not by study volume. For example, a single cohort evaluated under two pathology protocols yielded sensitivity estimates differing by 32 percentage points; pooling them would average across incompatible diagnostic scales. Studies are grouped by clinical setting to reflect the distinct diagnostic anatomy of intact versus previously operated stomachs. As a post hoc, exploratory analysis that was not pre-specified in the protocol, we additionally pooled sensitivity across the treatment-naive cohorts examined by paraffin section of all regional nodes, using a DerSimonian–Laird random-effects model applied to the logit-transformed proportions and back-transformed to the sensitivity scale (meta package, https://CRAN.R-project.org/package=meta, accessed on 11 October 2026; R version 4.3.1). This analysis is reported only to bound the plausible range of sensitivity in that subgroup and does not supersede the qualitative synthesis.

## 3. Results

### 3.1. Study Selection

The searches yielded 3142 records from five bibliographic databases and 38 from two trial registries. After removing 1608 duplicates, 1572 unique records were screened by title and abstract, leading to the exclusion of 1512. Sixty-one full-text reports were assessed: 60 retrieved from databases and registries, and one identified via citation searching. Seventeen reports corresponding to 11 unique studies met all eligibility criteria and were included, along with two ongoing trial records. Eight cohorts provided per-patient diagnostic accuracy data, three studies contributed four per-node molecular comparisons, and one randomized trial (along with its secondary and post hoc reports) provided the outcome stream.

### 3.2. Study Characteristics

The included cohorts span two decades of practice and encompass the four technical eras summarized in Table 1: dye read by eye, radioisotope combined with dye, lymphatic basin dissection, and near-infrared fluorescence. Their study-level characteristics, including design, setting, population, analyzed sample size, tracer strategy, index test, and reference standard, are detailed in Table 2. Six cohorts enrolled treatment-naive patients, while two enrolled patients after non-curative endoscopic resection.

Eight cohorts reported per-patient accuracy in a format that allowed contingency counts to be recovered; their characteristics are presented in Table 2, their recalculated performance in Table 3 (complete 2 × 2 counts in Table 4), and risk-of-bias judgments for all eight in Section 3.10. The patient serves as the unit of analysis, with histopathology of the regional nodes as the reference standard. A patient with clear sentinel nodes but metastasis elsewhere counts as a false negative.

The two settings (unoperated vs. previously operated stomachs) are treated separately below, as mapping an unoperated stomach presents different challenges than mapping one where submucosal scarring may have redirected lymphatic flow.

### 3.3. Detection Rates

Detection rates only loosely correlate with the mapping technique. Six of the eight entries in Table 3 range from 97.1% to 100%, spanning cohorts across two decades, 44 to 397 patients, and both single-center and multicenter consortia. The two rates below 95% are from the naked-eye dye arm of Takahashi’s trial [21] and Shoji’s 20-patient series [28]. Tracer strategy does not explain this spread. For instance, JCOG0302 achieved 97.7% with ICG read by eye, whereas Shoji achieved 85.0% with fluorescence [26,28]. Takahashi also stratified seven surgeons by experience; basin detection was 100% in every stratum with no false negatives under infrared, while the same surgeons found sentinel nodes in only 79.5% of the same patients under ordinary light. This suggests that the imaging technique, rather than operator experience, was the larger factor, although the trial was too small to definitively exclude a learning effect.

### 3.4. Diagnostic Accuracy in Treatment-Naive Disease

**Figure 2:**
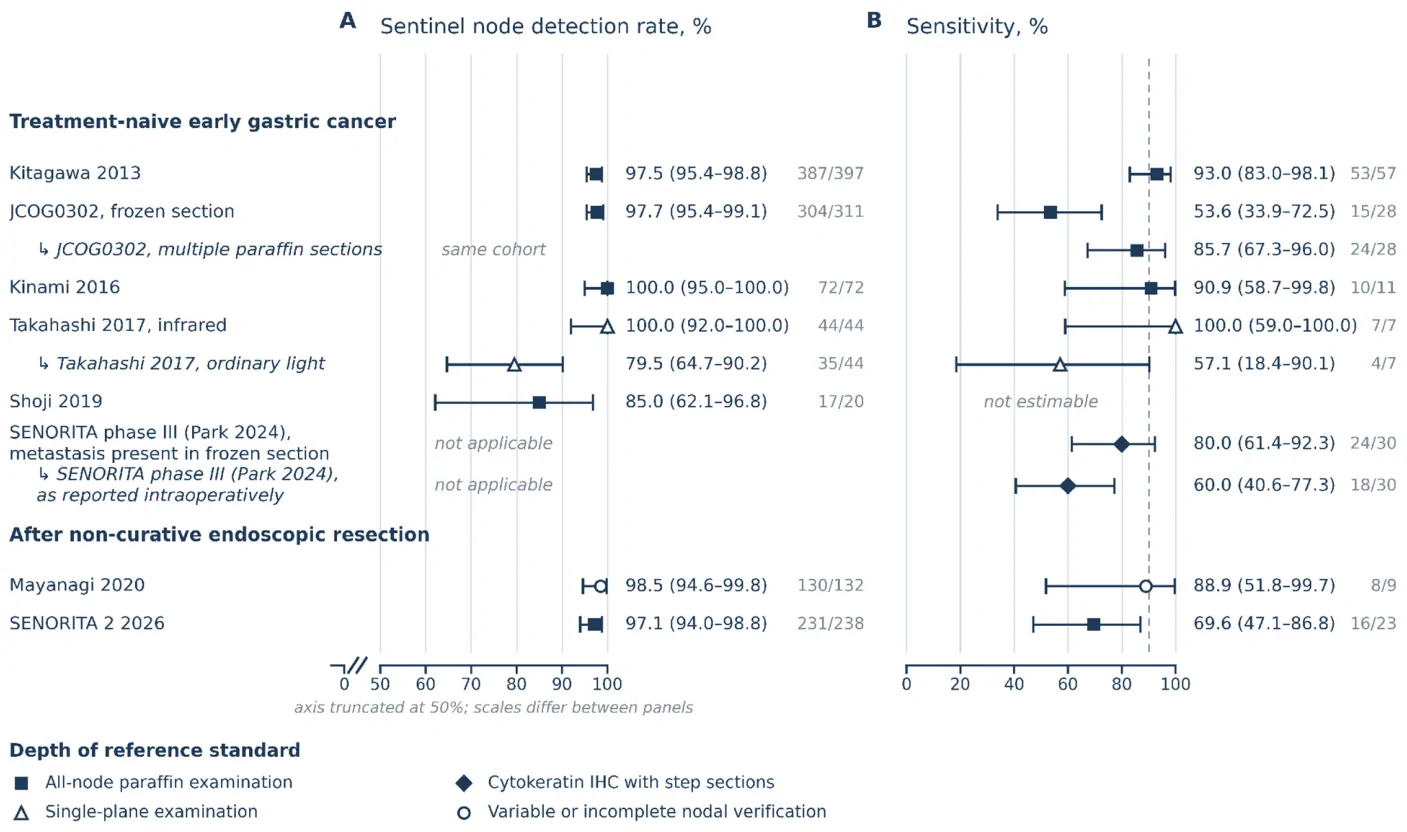
Per-patient detection rate and sensitivity, with exact binomial 95% confidence intervals. (**A**) Sentinel node detection rate; (**B**) sensitivity. Markers are point estimates and horizontal lines are Clopper–Pearson exact 95% confidence intervals, recalculated from the counts in each source report. Marker shape and fill denote the depth of the reference standard; marker size is uniform and encodes no weight, as no pooled estimate is computed. Indented italic rows (prefixed ↳) are the same cohort reanalyzed under a second index test, reading basis or pathology protocol. Source reports: Kitagawa 2013 [13]; JCOG0302 [26]; Kinami 2016 [27]; Takahashi 2017 [21]; Shoji 2019 [28]; SENORITA phase III (Park 2024) [19,29]; Mayanagi 2020 [9]; SENORITA 2 2026 [22,30]. The two panels use different horizontal scales, and panel A is truncated at 50%, so interval widths should not be compared by eye between panels. The full legend is given in the Supplementary Note S2.

In treatment-naive disease, the precision of accuracy estimates is limited by the number of node-positive patients available for analysis and by the depth of the reference standard against which they are counted, rather than by the mapping technique. Sentinel node detection is consistently high, exceeding 97% across institutions, but only Kitagawa’s cohort yields a usably narrow sensitivity interval. That phase II trial identified 53 out of 57 node-positive patients (93.0%; 95% CI, 83.0–98.1) [13]. JCOG0302 and SENORITA phase III accrued 28 and 30 node-positive patients, but were measured against different reference standards and yield intervals no narrower than 29 percentage points (Section 3.7). The remaining ICG series had fewer than twelve node-positive patients each, leaving confidence intervals too wide to confirm or refute mapping accuracy (Table 3 and Figure 2) [21,27,28].

The available estimates are also difficult to compare directly because they are measured on different scales. Sensitivity denominators vary, as most series only count patients with a detected node, while Takahashi counts all enrolled patients. Reference standards also differ in depth, meaning the figures reflect index test–reference standard pairings as much as the mapping itself (Section 3.7). JCOG0302 illustrates both effects within a single cohort: single-plane frozen section identified 15 out of 28 node-positive patients (53.6%), rising to 24 out of 28 (85.7%) when the same nodes were examined with multiple paraffin sections [26]. As a post hoc, exploratory analysis (Section 2.8), restricting synthesis to the treatment-naive cohorts examined by paraffin section of all regional nodes (Kitagawa, 53/57; the multiple-paraffin re-analysis of JCOG0302, 24/28; and the feasibility phase of Kinami, 7/7—a subset of the combined Kinami cohort of 10/11 reported in Table 3, whose single false negative arose in the frozen-section clinical phase), a random-effects model gives a pooled sensitivity of 89.4% (95% CI, 81.2–94.3; I^2^ = 0%); the lower confidence bound still permits roughly one node-positive patient in five to be missed, and this subgroup continues to combine single- and multiple-plane paraffin examination, so narrative synthesis remains the primary approach (Section 2.8).

### 3.5. Diagnostic Accuracy After Non-Curative Endoscopic Resection

Mapping is markedly less reliable after non-curative endoscopic resection, where submucosal scarring can redirect lymphatic flow. In the only prospective trial in this setting, SENORITA 2 (*n* = 238), mapping missed seven out of 23 node-positive patients (sensitivity 69.6%; 95% CI, 47.1–86.8), despite a 97.1% detection rate [22,30]. A smaller retrospective cohort reported higher sensitivity (Mayanagi, 88.9%; 8/9) [9]. However, its reference standard was incomplete and varied by patient, with approximately a quarter of patients receiving less than full regional clearance. Unexamined patients were counted as true negatives, inflating sensitivity by an unquantifiable amount. This cohort is the only one at high risk of bias across all four QUADAS-2 domains (Section 3.10). Sentinel-node mapping, sentinel-basin assessment, and per-node pathology are distinct endpoints that can diverge within one patient: in Mayanagi’s cohort, the single false negative was not a mapping failure but a metastasis in a non-sentinel node (station 5) lying within a correctly mapped basin, so a basin-level and a node-level analysis of the same case reach different conclusions.

In this setting, false negatives resulted from mapping rather than pathological examination. SENORITA 2 did not use frozen sections, and deeper sectioning of all seven false-negative nodes found no micrometastases. The investigators attributed these misses to post-resection fibrosis and skip metastasis, where the missed disease lay outside the mapped basin [22]. Due to small patient numbers and varying tracer choices across participating centers, tracer subgroups could not be compared, making it impossible to separate the effect of the tracer from that of the center. The evidence for mapping after endoscopic resection relies on a single feasibility-powered trial that missed approximately three out of every ten node-positive patients.

### 3.6. Mapping Compared with Anatomically Defined Regional Dissection

A post hoc analysis of 237 SENORITA navigation-arm patients investigated whether mapping offers advantages over anatomically defined dissection [19]. This analysis reconstructed the yield of regional lymphadenectomy performed without mapping from the same specimens. Sensitivity was 96.8% with mapping versus 80.6% without (*p* = 0.016), and the negative predictive value was 99.5% versus 97.2% (*p* = 0.021). Metastasis outside a tumor-free sentinel basin occurred in only one out of 237 patients (0.4%). Its node-positive denominator differs by one from the pathology re-review of the same 237 patients (Section 3.7), reflecting the different reference protocols of the two reports [19,29]. Because the comparator was reconstructed rather than randomized, this analysis is hypothesis-generating and applies only to treatment-naive disease.

### 3.7. Pathology Protocol Depth and Measured Sensitivity

Two within-cohort comparisons demonstrate that measured sensitivity is highly dependent on the thoroughness of node examination. In JCOG0302 [26], the same 311 patients and sentinel nodes yielded 53.6% sensitivity with single-plane frozen section versus 85.7% with multiple paraffin sections. Since the surgical procedure remained unchanged, the 32-percentage-point difference is solely attributable to the pathology protocol.

The SENORITA phase III cohort [29] identifies two ways a protocol can fail. Re-examination of all 237 navigation-arm patients revealed that 30 were node-positive on enhanced permanent pathology. Frozen section identified all 13 macrometastases but missed six out of 17 micrometastases. This allows for the calculation of two different sensitivities for the same 30 patients.

When measured against metastases identifiable on the frozen slide upon later review (permanent pathology), sensitivity was 80.0% (24/30; 95% CI, 61.4–92.3). However, when measured against the result of the frozen section reported to the surgeon during the operation, sensitivity was 60.0% (18/30; 40.6–77.3). The 20-percentage-point difference (six patients) represents cases where deposits were present on the slide examined by the pathologist but were not identified at the time, leading to a negative intraoperative report. The six-patient gap therefore reflects deposits present on the same frozen slide the pathologist examined but not reported during the operation (an interpretation difference), not additional tissue sampling, because no further sections were introduced between the intraoperative reading and the review.

This discrepancy highlights the difference between sampling failure and interpretation failure. Sampling failure occurs when a deposit lies outside the plane that was cut and can be recovered by deeper sectioning. Interpretation failure, conversely, is when a deposit present on the slide is not recognized. Deposit size influences both types of failure; a protocol adequate for macrometastasis may still miss micrometastatic disease. Notably, none of the six patients with missed micrometastases developed nodal recurrence, leading the authors to conclude that single-section examination is adequate in practice [29]. Therefore, an argument for a deep intraoperative protocol has been made based on avoiding under-staging rather than on a demonstrated reduction in recurrence. A separate prospective cohort in treatment-naive early gastric cancer reinforces this: its measured false-negative rate fell from 18.2% with intraoperative hematoxylin–eosin alone to 0% once rapid cytokeratin immunohistochemistry was added, confirming that sentinel-node sensitivity reflects the depth of the pathology protocol as much as the mapping itself [31].

However, the depth of pathological examination does not fully account for all instances of low sensitivity. For example, SENORITA 2 did not utilize frozen sections and found no micrometastases even after deeper sectioning of all seven false-negative nodes. This suggests that its failures were related to the mapping technique itself [22]. Kinami’s single false-negative case was attributed to frozen-section failure [27]. In contrast, Takahashi’s 100% sensitivity [21] was measured against a shallow, single-plane standard. This method is less likely to reveal a false negative compared to the all-node paraffin examination employed by Kitagawa, JCOG0302, and Kinami [13,26,27].

Thus, reported sensitivity reflects the specific combination of the index test and the reference standard, rather than the mapping technique in isolation. This, more than the limited number of studies, is the primary reason why no pooled estimate is offered. Any center adopting SNNS must address both the pathology protocol and the operative technique. These within-cohort comparisons establish a strong pathology effect within the cohorts studied; they do not, however, establish that pathology contributes more than mapping to the discordance seen across the literature as a whole.

### 3.8. Molecular Nodal Assessment

Intraoperative pathology is the principal limitation because the extensive depth of examination required for high sensitivity cannot be achieved within the operative window. One-step nucleic acid amplification (OSNA) offers a solution by quantifying cytokeratin 19 messenger RNA in homogenized nodal tissue within this timeframe [23]. By analyzing the entire node rather than sampling planes, OSNA avoids the sampling failure observed in JCOG0302 [25,26].

However, current validation does not yet support OSNA for this role. Three studies contributed four node-level comparisons against paired histopathology, revealing a consistent pattern (Table S2) [23,24,25]. OSNA rarely incorrectly identifies a negative node as positive, demonstrating specificity ranging from 95.9% to 99.5%. Its sensitivity, however, is less consistent, varying from 63.6% to 88.9%. The lowest sensitivity value comes from the only cohort restricted to early gastric cancer [25]; in those 43 patients, sensitivity was 63.6% against permanent sections and 80.0% against intraoperative sections.

Applicability to early disease is further limited: many tumors in the Yaguchi and Kumagai studies were advanced (17 out of 32 subserosal or serosa-exposed; 29 out of 61 T3 or T4), which tend to have larger deposits [23,24]. Discordance is concentrated in poorly differentiated and signet-ring histology [24,25], and homogenized tissue cannot be subsequently examined histologically [23]. These per-node figures establish agreement between the assay and the pathologist for a single node, but they do not confirm whether a tumor-free basin predicts a tumor-free field. OSNA is therefore reasonable as an adjunct to, rather than a replacement for, the standard pathology protocol. Patient-level validation in early disease is still lacking. These per-node binomial intervals also treat nodes as independent and therefore ignore clustering within patients, which can overstate precision; OSNA and histology are moreover performed on different physical portions of the same node (tissue-allocation bias), and paired histopathology is itself an imperfect reference rather than an error-free standard.

### 3.9. Clinical and Functional Outcomes

Randomized evidence from the SENORITA trial (580 patients across seven Korean centers) suggests a trade-off rather than a decisive advantage for either approach. Stomach-preserving navigation surgery did not establish non-inferiority for three-year disease-free survival (a 3.7% difference favoring standard gastrectomy; 95% CI, −0.6 to 8.1, spanning both the 5% margin and zero). This renders the trial inconclusive rather than negative [12]. While overall and disease-specific survival showed no statistically significant difference at five years (OS *p* = 0.7403; DSS *p* = 0.9586), effective endoscopic and surgical salvage of metachronous lesions may have contributed to this convergence rather than demonstrably superior nodal clearance, although the trial design cannot establish that mechanism. Additionally, later estimates for the standard-gastrectomy arm are based on short follow-up [32]. Serious complications were similar in both arms, indicating that adding mapping did not increase operative risk (Table 5) [33].

In exchange for intensive surveillance, patients who retained their stomach experienced functional benefits: quality of life was better on all eight scales at three months, and nutritional parameters were higher at one year (Table 5) [34]. These represent the program’s most consistent benefits, although the endpoints were patient-reported in an open-label trial. This carries a high risk of measurement bias under RoB 2 [18] and applies only to the 81.4% of the navigation arm who retained their stomach; the remaining patients converted to gastrectomy due to failed mapping, positive nodes, or inadequate margins [33]. Because this entire clinical and functional evidence base derives from a single phase III program conducted at seven Korean centers, these findings should be regarded as trial- and setting-specific rather than generalizable without confirmation elsewhere.

### 3.10. Risk of Bias and Applicability

Table 6 summarizes the strength of the evidence in each domain along with its primary limitations. Study-level QUADAS-2 judgments appear in Table 7 for per-patient cohorts and Table S3 for per-node comparisons. Eight cohorts contributed per-patient accuracy data, and all eight are appraised in Table 7: the seven independent diagnostic cohorts together with the SENORITA phase III diagnostic cohort, which is appraised in the final row. None of the appraised cohorts was at low risk of bias across all four domains. Mayanagi’s retrospective cohort was the only one rated high in every domain, mainly because the extent of lymphadenectomy—and thus the reference standard—varied between patients. SENORITA 2 carried the lowest risk among the post-endoscopic-resection cohorts, and the JCOG0302 frozen-section comparison was low in three of the four domains.

## 4. Discussion

### 4.1. Evolution and Technique of Sentinel Node Mapping

Adapted from breast cancer and melanoma protocols in the 1990s, sentinel node mapping encountered specific challenges in the stomach: multidirectional drainage, perigastric fat that obscures targeted nodes, and marked operator dependence [10,11,14]. Successive technical advancements, summarized in Table 1, have largely overcome these obstacles.

Early dye-only mapping, while inexpensive and requiring no equipment, was limited by a narrow timing window and poor visibility in fat [11]. Radioisotope mapping with technetium-99m tin colloid, injected submucosally the day before surgery, is retained in first-echelon nodes and can be localized by gamma probe regardless of overlying tissue. This technique, combined with dye, became the reference in the 2000s and underpinned Kitagawa’s trial [13]. However, its adoption remains constrained by the need for nuclear-medicine infrastructure and radiation-safety licensing [13,20].

Lymphatic basin dissection removes the primary vascular basin en bloc, ensuring that any metastasis missed by mapping still lies within the resected tissue [14,19]. The SENORITA post hoc analysis supports this, with metastasis lying outside a tumor-free basin in only one out of 237 patients [19]. In contrast, in JCOG0302, seven out of 13 false negatives had metastases outside the basin, a difference explained by that trial’s dye-only mapping, node-by-node retrieval, and single-plane examination [26].

Near-infrared fluorescence with indocyanine green (ICG) overcomes the tissue-penetration limits of visual dye without requiring radioisotopes. Takahashi’s within-patient comparison achieved 100% detection with infrared versus 79.5% under white light (Table 3) [21]. However, it has not yet displaced dual-tracer mapping due to the confounded, staggered adoption in SENORITA 2 and the small size of single-agent feasibility cohorts. Therefore, a well-powered randomized comparison of single-agent fluorescence and dual tracers is still needed [20,22,28].

### 4.2. Interpretation of the Randomized Evidence

At five years, disease-free survival did not differ significantly between the arms (88.9% versus 80.1%, *p* = 0.0561); this borderline result is fragile: follow-up in the standard-gastrectomy arm reached only 62.5 months, and with only about 24 patients per arm still at risk at 60 months (versus 254 and 229 at 54 months in the full analysis set) the standard-arm estimate is driven by few events and carries a wide confidence interval of 47.2 to 94.1 (Table 5) [32]. Overall and disease-specific survival showed no statistically significant difference (*p* = 0.7403 and 0.9586), and three-year disease-free survival favored standard gastrectomy [12]. The mechanism is more informative than any of these figures. Disease-specific survival may have been supported by effective surgical salvage (the early detection and resection of recurrences and metachronous lesions in the remnant stomach) rather than by superior mapping accuracy. Long-term success therefore depends on rigorous surveillance and timely salvage. This reliance on surveillance is itself a burden borne by the patient: it presumes sustained, potentially indefinite endoscopic follow-up, with cumulative procedural and financial cost, the possibility of declining adherence over time, and a psychological impact of ongoing cancer surveillance that the trial did not measure.

### 4.3. Current Role and Patient Selection

Current evidence does not establish SNNS as the standard of care but supports its selective use at high-volume centers. For treatment-naive early gastric cancer meeting SENORITA criteria, SNNS offers acceptable diagnostic accuracy and superior functional preservation, with no statistically significant difference in five-year overall or disease-specific survival and a five-year disease-free survival difference that did not reach significance, despite the unmet non-inferiority endpoint [13,32,34]. Until a trial succeeds, SNNS should be confined to clinical trials and adequately resourced centers, and offered only to patients who are counseled that gastric preservation is likely but not guaranteed. No universally accepted false-negative threshold exists for gastric sentinel node surgery; the benchmarks commonly adapted from breast cancer, melanoma, and the Japanese trials are a false-negative rate of 10% or less (sensitivity of 90% or more) together with a detection rate of at least 95%. Even the largest treatment-naive cohort meets this only at its point estimate: its lower 95% confidence bound of 83% still permits a clinically important rate of missed nodal disease. Selective use should therefore be confined to the criteria under which it has been studied—clinical T1, cN0, solitary tumors of 4 cm or less, located away from sites that would otherwise require total or proximal gastrectomy.

There is currently insufficient evidence to support omitting standard lymphadenectomy after non-curative endoscopic resection. A prospective multicenter trial reported 69.6% sensitivity, missing seven out of 23 node-positive patients [22]. The higher accuracy suggested by retrospective data [9] was subject to verification bias from an incomplete reference standard, which inflates sensitivity by an amount the published data cannot quantify. Omitting lymphadenectomy in this setting cannot be recommended [22].

Benefit also depends on the planned resection: patients facing total or proximal gastrectomy gain more from preservation than those undergoing distal resection [3,34]. Not every preserving operation maintains function equally, as segmental resection was linked to diarrhea and eating restriction at three years [34].

### 4.4. Technical Requirements

The clinical adoption of SNNS hinges on adherence to three core technical standards:

1. **Tracer selection.** Available evidence favors dual-tracer or near-infrared fluorescence mapping for reliable sentinel node detection, whereas single-agent visual dye has shown lower detection when compared within a trial [20,21]; its effect on sensitivity, however, remains unestablished (Table 6). Dual-tracer or fluorescence mapping is therefore preferred, although it has not been shown to improve sensitivity.
2. **Resection unit.** Intraoperative removal must encompass the entire lymphatic basin as a single unit, rather than involving selective node-by-node excision [14,19].
3. **Standardized pathology protocols.** Institutions must establish a sectioning and staining protocol prior to implementation, as measured sensitivity is highly dependent on pathological depth (Section 3.7). While deeper sectioning corrects for sampling error, it does not address interpretation error. Standardized processing is a prerequisite for adoption.

### 4.5. Limitations

The existing literature presents both geographic and methodological limitations. Most diagnostic cohorts originate from Japanese and Korean centers, which identify a high proportion of early-stage disease through screening. Consequently, the generalizability of these findings to Western populations, and to other Asian populations such as Chinese patients, remains unproven; these groups differ in screening exposure, tumor biology, baseline stage distribution, and accumulated mapping experience [1]. Node-positive events are rare in most cohorts, which restricts the precision of sensitivity estimates. In SENORITA 2, 73.5% of patients were recruited at the lead center [22]. The depth of reference standards also varied across cohorts.

Review-level limitations include retrospective protocol registration, restriction to English-language publications, a major limitation that may bias the evidence base (this excluded four full-text articles [35,36,37,38]; Figure 1), and the inability to contact authors directly for missing data. A quantitative assessment of publication bias was not possible, as Deeks’ test [39] requires at least ten studies [40]. Because fewer than ten cohorts contributed, we cannot statistically exclude the selective non-publication of small feasibility studies with poor mapping results; any such bias would inflate reported detection rates more than sensitivity estimates.

One appraisal finding pertains to the field as a whole rather than to individual studies: none of the eight appraised cohorts achieved a low risk of bias across all four QUADAS-2 domains [15]. This is primarily because the index test and reference standard are not independent in surgical mapping (Section 2.7), meaning that reported accuracy addresses a narrower question than it might appear to.

### 4.6. Future Directions

Establishing the oncological safety of mapping after non-curative endoscopic resection necessitates a trial powered for diagnostic accuracy rather than mere feasibility. Such a trial would need to enroll a sufficient number of node-positive patients to meaningfully define sensitivity, under a pathology protocol prespecified for both arms rather than left to individual centers. No trial has yet met either requirement.

Two registered trials aim to address these gaps. A Japanese multicenter non-randomized phase III trial [41] plans to enroll 225 patients across 13 centers. This trial will compare stomach preservation with standard gastrectomy using dual-tracer mapping, intraoperative pathology, and basin dissection in sentinel-negative patients, with five-year relapse-free survival as the primary endpoint. Its design differs sufficiently from SENORITA that a concordant result would carry considerable weight. A Korean feasibility study of fluorescence-guided mapping is also registered [42].

Reporting standards should improve in one key area: studies should present complete two-by-two contingency tables alongside the sectioning and staining protocol for both sentinel and non-sentinel nodes. This would allow readers to directly verify true-positive and false-negative rates, rather than relying on opaque denominators. Adhering to STARD reporting guidelines [43] would achieve this. Since detection and sensitivity are diagnostic accuracy endpoints, STARD naturally complements the surgical reporting standards, such as STROCSS [44], that studies in this field commonly follow [22].

## 5. Conclusions

Sentinel node navigation surgery reliably identifies sentinel nodes but is less consistent in ruling out nodal disease. Within the cohorts that examined it, this variability was linked at least as much to the depth of pathological examination as to the mapping technique, highlighting that the pathology protocol warrants as much scrutiny as the tracer.

For treatment-naive early gastric cancer, the accuracy evidence, although largely based on a single adequately powered cohort, supports stomach preservation within clinical trials and at adequately resourced centers. This approach offers improved function and quality of life in exchange for intensive surveillance, and patients should be counseled that gastric preservation is likely but not guaranteed.

In contrast, after non-curative endoscopic resection, the current evidence does not support omitting lymphadenectomy. Broader adoption should await trials powered for diagnostic accuracy and reported with complete contingency data.

## Figures and Tables

**Table 1:** Technical evolution of sentinel node mapping in gastric cancer.

Era	Technique	Problem Addressed	Limitation Exposed	Key Studies
Late 1990s Single dye	Peritumoral dye injection ICG read as green stain by eye Later isosulfan or patent blue Node identified by inspection	Whether the sentinel concept transfers to an organ with multidirectional drainage	Narrow timing window, poor visibility in fat, operator-dependent, and contested from the outset [10]	Hiratsuka 2001 [11]
2000–2010 Dual tracer	99mTc tin colloid, endoscopic submucosal injection, 4 quadrants, day before Gamma probe Combined with dye	Short dye residence time Poor visibility in fat Large particle size retained in first-echelon nodes	Requires nuclear-medicine facility and isotope licensing, plus extra preoperative endoscopy	Kitagawa 2013 [13] detection 97.5%
2008 onward Basin dissection	Unit of resection = basin, not node Five basins by feeding artery En bloc removal Nodes identified ex vivo	Skip metastasis Unreliability of any single node	Conceptual rather than diagnostic; enlarges the safety margin without improving sensitivity	Kinami 2008 [14] SENORITA post hoc [19]: 1/237 (0.4%) outside basin
2010–present Fluorescence	ICG viewed with infrared or near-infrared imaging Infrared endoscopy or laparoscopy No isotope required	Dye invisible through fat Infrastructure burden of radioisotope	Has not matched dual tracer when compared within a trial; cameras and ICG dose not standardized [20]	Takahashi 2017 [21]: 100% vs. 79.5% SENORITA 2 [22]: 97.7% vs. 90.9%
Present Intraoperative assay	OSNA on nodal tissue Laparoscopic and robotic platforms	Intraoperative pathology as the weakest link (JCOG0302)	Validated per node, not per patient; mostly non-early-gastric-cancer cohorts	Yaguchi 2011 [23] Kumagai 2014 [24] Shimada 2020 [25]

**Legend**: Eras overlap. Dates mark general use in the cohorts of Table 2 and Table 3, not first description. ICG appears in the first and fourth eras: it entered practice as a dye read by eye and returned as a fluorescence tracer. The agent did not change; the detection method did. ICG, indocyanine green; OSNA, one-step nucleic acid amplification; 99mTc, technetium-99m.

**Table 2:** Study characteristics, per-patient diagnostic accuracy cohorts.

Study	Design	Population	*N*	Tracer	Index Test	Reference Standard
**Treatment-naive early gastric cancer**						
Kitagawa 2013 [13] Japan	Prospective multicenter Single-arm phase II	Treatment-naive	397	Dual 99mTc-tin colloid + isosulfan blue	Hot and/or blue node Paraffin	Final paraffin All nodes
JCOG0302 2014 [26] Miyashiro, Japan	Prospective multicenter phase II Accrual suspended	Treatment-naive	311	Single: ICG	Green node Frozen section, one plane	Final paraffin All nodes
*↳ JCOG0302, re-examined* *same cohort*	Second pathology protocol applied to the same nodes	Treatment-naive	311	Single: ICG	Green node Multiple paraffin sections	Final paraffin All nodes
Kinami 2016 [27] Japan	Prospective feasibility Single center, two phases	Treatment-naive	72	Single: ICG fluorescence	Bright node on fluorescence	Final paraffin All nodes
Takahashi 2017 [21] infrared, Japan	Prospective multicenter	Treatment-naive	44	Single: ICG Infrared ray laparoscopy	Node visible under infrared light	Final paraffin One plane
*↳ Takahashi 2017* [21] *ordinary light, same cohort*	Comparator index test in the same patients	Treatment-naive	44	Single: ICG Naked-eye observation	Green node under ordinary light	Final paraffin One plane
Shoji 2019 [28] Japan	Prospective feasibility Single center	Treatment-naive 2 of 20 pT2 on final pathology	20	Single: ICG fluorescence + OSNA	Fluorescent node OSNA	OSNA Final paraffin
SENORITA phase III (Park 2024) [29] Park, Korea	Retrospective analysis of a randomized trial Sentinel basin nodes of the navigation arm	Treatment-naive	237	Dual RI + ICG	Intraoperative frozen section Single plane, HE	FFPE with cytokeratin IHC Three 200 µm step sections
*↳* *SENORITA phase III, as reported intraoperatively* *same cohort*	Second reading basis applied to the same nodes	Treatment-naive	237	Dual RI + ICG	Frozen section report issued during surgery	FFPE with cytokeratin IHC Three 200 µm step sections
**After non-curative endoscopic resection**						
Mayanagi 2020 [9] Japan	Multicenter retrospective 2001–2016	Post-endoscopic resection	132	Dual, ~52% Single, ~48%	Hot node on gamma probe Stained or fluorescing node	Final paraffin Lymphadenectomy extent varied
SENORITA 2 2026 [22,30] Park, Korea	Prospective multicenter Phase II feasibility	Post non-curative ESD	238	Dual RI + ICG, 90.8% ICG alone, 9.2%	Sentinel basin node No frozen section	Final paraffin After D1+ gastrectomy

**Legend**: Rows prefixed ↳ and set in italics are the same patient cohort reanalyzed under a different index test or pathology protocol, not independent studies. JCOG0302 contributes two rows and Takahashi 2017 [21] contributes two. Ref. [22] SENORITA 2 results paper; Ref. [30] trial protocol. The SENORITA 2 phase II trial is distinct from the SENORITA phase III trial reported in Section 3.9. ESD, endoscopic submucosal dissection; ICG, indocyanine green; OSNA, one-step nucleic acid amplification; RI, radioisotope.

**Table 3:** Per-patient diagnostic performance.

Study	Detection % (*n*/*N*) [95% CI]	pN+ (n)	Sensitivity % (*n*/*N*) [95% CI]	NPV % (*n*/*N*) [95% CI]
**Treatment-naive early gastric cancer**				
Kitagawa 2013 [13]	97.5 (387/397) (95.4–98.8)	57	93.0 (53/57) ^†^ (83.0–98.1)	98.8 (330/334) (97.0–99.7) a
JCOG0302 [26] frozen section	97.7 (304/311) (95.4–99.1)	28	53.6 (15/28) ^†^ (33.9–72.5)	95.5 (274/287) (92.4–97.6) b
*↳ JCOG0302* [26] *multiple paraffin sections*	97.7 (304/311) (95.4–99.1)	28	85.7 (24/28) ^†^ (67.3–96.0)	NR
Kinami 2016 [27]	100.0 (72/72) (95.0–100.0)	11	90.9 (10/11) ^†^ (58.7–99.8)	98.4 (61/62) (91.3–100.0)
Takahashi 2017 [21] infrared	100.0 (44/44) (92.0–100.0)	7	100.0 (7/7) ^‡^ (59.0–100.0)	100.0 (37/37) (90.5–100.0)
*↳ Takahashi 2017* [21] *ordinary light*	79.5 (35/44) (64.7–90.2)	7	57.1 (4/7) ^‡^ (18.4–90.1)	92.5 (37/40) (79.6–98.4) c
Shoji 2019 [28]	85.0 (17/20) (62.1–96.8)	1	NE	NE
SENORITA phase III [19,29] (Park 2024) metastasis present in frozen section	NA	30	80.0 (24/30) ^§^ (61.4–92.3)	NR
*↳ SENORITA phase III (Park 2024)* *as reported intraoperatively*	NA	30	60.0 (18/30) ^§^ (40.6–77.3)	NR
**After non-curative endoscopic resection**				
Mayanagi 2020 [9]	98.5 (130/132) (94.6–99.8)	9	88.9 (8/9) ^†^ (51.8–99.7)	99.2 (121/122) (95.5–100.0)
SENORITA 2 2026 [22,30]	97.1 (231/238) (94.0–98.8)	23	69.6 (16/23) ^†^ (47.1–86.8)	96.7 (208/215) (93.4–98.7)

**Legend**: Unit of analysis: patient. Exact binomial (Clopper–Pearson) 95% CIs were recalculated from published counts. Rows prefixed ↳ and set in italics are the same cohort reanalyzed under a second index test or protocol, not independent studies. Denominators are not uniform: ^†^ patients with a detected sentinel node; ^‡^ all enrolled patients (intention-to-diagnose; Takahashi); ^§^ the analyzed cohort, in whom basin dissection had already succeeded (SENORITA phase III). The SENORITA phase III reference standard, cytokeratin immunohistochemistry with three 200 µm step sections, is deeper than the all-node paraffin examination used elsewhere, so those rows are not on a common scale with the others. Footnotes: a, Kitagawa true-negative count reconstructed from totals; b, JCOG0302 frozen section includes two frozen-positive calls negative on final paraffin; c, Takahashi accuracy computed across all 44 enrolled patients. CI, confidence interval; NA, not applicable; NE, not estimable; NPV, negative predictive value; NR, not reported; pN+, node-positive. Kinami 2016 [27] combined a feasibility phase (*n* = 42; D1+/D2 with permanent examination of all nodes) and a clinical phase (*n* = 30; ICG lymphatic-basin dissection with intraoperative frozen section), and the single false negative arose in the frozen-section clinical phase. Takahashi’s NPV (37/40) is an intention-to-diagnose value in which the nine ordinary-light mapping failures are counted as test-negative; rows are otherwise reported on the detected-node (evaluable) basis.

**Table 4:** Complete 2 × 2 contingency counts and mapping failures for each per-patient analysis. FP = 0, where the sentinel node and reference standard derive from the same paraffin examination; JCOG0302 multiple-paraffin TN was not reported (NR); Shoji is not estimable (NE; 1 pN+). Counts are on the detected-node basis except Takahashi (intention-to-diagnose).

Analysis (Table 3 Row)	TP	FP	FN	TN	Mapping Failures (*n*/*N*)
Kitagawa 2013 [13]	53	0	4	330	10/397
JCOG0302, frozen section [26]	15	2	13	274	7/311
JCOG0302, multiple paraffin [26]	24	0	4	NR	7/311
Kinami 2016 [27]	10	0	1	61	0/72
Takahashi 2017, infrared [21]	7	0	0	37	0/44
Takahashi 2017, ordinary light [21]	4	0	3	37	9/44
Shoji 2019 [28]	NE	NE	NE	NE	3/20
Mayanagi 2020 [9]	8	0	1	121	2/132
SENORITA 2 2026 [22,30]	16	0	7	208	7/238

**Table 5:** SENORITA phase III trial outcomes.

Report	Endpoint	Analyzed	Result	Interpretation
An 2020 Br J Surg [33]	Short-term morbidity Secondary endpoint	527 in modified ITT LSG 269, LSNNS 258	Clavien–Dindo ≥ III LSG, 16/269 (6%) LSNNS, 13/258 (5%)	No difference in serious complications Morbidity of mapping itself cannot be isolated
Kim 2022 J Clin Oncol [12]	3-year DFS Primary endpoint	580 randomly assigned LSG 292, LSNNS 288 Median follow-up: 47.5 mo	3 y DFS, 95.5% vs. 91.8% Difference, 3.7% (95% CI, −0.6 to 8.1) Margin, 5% HR, 1.901 (0.911–3.967)	Non-inferiority not demonstrated CI crosses margin and zero Inconclusive, not negative
Eom 2024 JAMA Surg [34]	QoL and nutrition Prespecified secondary	194 preserved vs. 257 standard 7 Korean centers Mean age: 55.6	At 3 mo, favoring preservation: physical function, 87.2 vs. 83.9; eating restriction, 10.9 vs. 18.2; appetite loss, 13.1 vs. 19.4; dysphagia, 8.0 vs. 12.7; dyspnea, 5.9 vs. 11.2; anxiety, 29.0 vs. 35.2; taste change, 7.4 vs. 13.0; body image, 19.5 vs. 27.2 At 1 y: BMI, 23.9 vs. 22.1; hemoglobin, 14.3 vs. 13.3 g/dL; albumin, 4.3 vs. 4.25 g/dL; protein, 7.3 vs. 7.1 g/dL Segmental resection associated with diarrhea and eating restriction at 3 y	Most consistent advantage for preservation Open-label, self-reported Segmental resection is the weaker preserving operation
Hur 2025 Ann Surg [32]	5-year DFS, OS, DSS	527 in modified ITT LSG 269, LSNNS 258 Median follow-up ~59 mo	5 y DFS, 80.1% (47.2–94.1) vs. 88.9% (84.0–92.3), *p* = 0.0561 OS *p* = 0.7403; DSS *p* = 0.9586 Per protocol: LSG 80.6% (47.2–94.0) significantly lower than LSNNS 88.2% (83.2–91.9) Stomach preserved 198 analyzed for local control; 210/258 (81.4%) had stomach-preserving surgery [33]	Estimates now favor navigation, reversing the 3-year direction LSG estimate unstable: follow-up in that arm reached only 62.5 months, so few patients remained at risk at 5 y 10/16 LSG events were second primaries in other organs vs. 13/27 gastric-related after LSNNS Investigators conclude navigation surgery could be recommended as an alternative

**Legend**: All four reports draw on the same randomized trial. Values reproduced as published, not recalculated, unlike Table 3 and Table S2. Two further reports are diagnostic rather than outcome papers and are not tabulated: Park SH 2024 (Ann Surg Oncol) [19] (Section 3.6) and Park SH 2024 (Gastric Cancer) [29] (Section 3.7). Distinct from the SENORITA 2 phase II trial (Table 2 and Table 3), which enrolled patients after non-curative endoscopic resection. QoL scores are EORTC scales; higher is better for functional scales, lower is better for symptom scales. DFS, disease-free survival; DSS, disease-specific survival; HR, hazard ratio; LSG, laparoscopic standard gastrectomy; LSNNS, laparoscopic sentinel node navigation surgery; OS, overall survival; QoL, quality of life.

**Table 6:** Critical appraisal by evidence domain.

Domain	Studies	Evidence	Limitations	Verdict
Detection	All per-patient cohorts	97.1–100% in 6 out of 8 entries 79.5% naked-eye dye (Takahashi) 85.0% fluorescence, 20 patients (Shoji) 97.7% single ICG read by eye (JCOG0302)	Almost all Japanese and Korean centers No learning-curve effect across 7 surgeons, but on 44 patients	Reliable. Tracer does not fully account for the spread; the two dye results are not reconciled.
Accuracy, treatment-naive	Kitagawa; JCOG0302; Kinami; Takahashi; Shoji	Sensitivity, 93.0% (83.0–98.1), Kitagawa, *n* = 397, 57 pN+ Supporting series much smaller	Only Kitagawa’s interval is usably narrow (15 points) JCOG0302 28 pN+ but interval ≥ 29 points; Kinami and Takahashi, 7–11 pN+ Shoji not estimable (1 pN+) SENORITA phase III appraised under pathology protocol depth	Acceptable in the largest cohort. Unquantified elsewhere.
Accuracy, post-ESD	SENORITA 2; Mayanagi	SENORITA 2 prospective, *n* = 238; sensitivity, 69.6% Mayanagi retrospective, *n* = 132; sensitivity, 88.9%	Overlapping CIs, opposing estimates Mayanagi: 9 pN+, 15-year accrual, variable reference standard SENORITA 2: 73.5% at lead center; no frozen section, so failure is mapping Attributed to post-ESD fibrosis and skip metastasis	Not supported. Insufficient to omit lymphadenectomy.
Pathology protocol depth	JCOG0302; SENORITA phase III; Kinami	53.6% → 85.7% in the same 311 patients when frozen section was replaced by multiple paraffin sections 80.0% vs. 60.0% in the same 237 SENORITA phase III patients, separating sampling failure from interpretation failure Kinami’s single false negative attributed to frozen-section failure	Few cohorts report two protocols No prospective study designed to test it Does not explain SENORITA 2	Determines the estimate. Sensitivity describes an index test–reference standard pairing; principal reason for no pooled estimate.
Tracer strategy	Takahashi; Mayanagi; SENORITA 2	Within-cohort: infrared vs. ordinary light, 100% vs. 79.5% in the same 44 patients SENORITA 2: dual, 97.7%/70.0%; fluorescence, 90.9%/66.7%	SENORITA 2 subgroups confounded by center Denominators too small (authors’ own view) Mayanagi used mixed tracers No randomized comparison	Dual tracer favored for detection. Effect on sensitivity unestablished.
Mapping vs. regional dissection	SENORITA post hoc [19]	Post hoc, *n* = 237 Sensitivity, 96.8% vs. 80.6% (*p* = 0.016) NPV, 99.5% vs. 97.2% (*p* = 0.021) 1/237 (0.4%) outside a tumor-free basin	Post hoc and hypothesis-generating Comparator reconstructed, not randomized Treatment-naive only	Hypothesis-generating. Suggests added value over anatomical regional dissection.
Molecular assessment	Yaguchi; Kumagai; Shimada	Four node-level comparisons from three studies, against paired histopathology Specificity, 95.9–99.5% Sensitivity, 63.6–88.9%	Per node, not per patient Yaguchi and Kumagai are not early gastric cancer cohorts Concordance dominated by negative nodes Discordance concentrates in poorly differentiated and signet-ring histology	Adjunct only. Not a standalone intraoperative reference standard.
Oncological outcomes	SENORITA phase III	Randomized trial with 5-year follow-up 3 y DFS 95.5% vs. 91.8% No significant 5 y difference	Non-inferiority not shown: 3.7%; CI, −0.6 to 8.1 vs. 5% margin Open-label Survival equalized by salvage, not nodal accuracy	Inconclusive, not negative. Commits a center to surveillance.
Morbidity	SENORITA phase III	Clavien–Dindo ≥ III, 6% vs. 5%	Heterogeneous definitions Mapping morbidity not separable from resection Single randomized trial	No signal. Adding mapping did not raise the complication rate.
Function and nutrition	SENORITA phase III	Prespecified secondary analysis 194 preserved vs. 257 standard Better QoL at 3 mo, better BMI and hematology at 1 y	Single trial Open-label with self-reported endpoints	Favors preservation. The most robust advantage the program has shown.

**Legend**: Studies are named by first author or trial; full citations are in Table 2, Table 3, Table 4 and Table 5 and the reference list. SENORITA phase III comprises six reports (An 2020 [33], Kim 2022 [12], Eom 2024 [34], two Park SH 2024 papers [19,29], Hur 2025 [32]); it is distinct from the SENORITA 2 phase II trial [22,30]. Study-level risk-of-bias judgments are given in Table 7 and, for the per-node comparisons, Table S3. CI, confidence interval; DFS, disease-free survival; ESD, endoscopic submucosal dissection; FNR, false-negative rate; NPV, negative predictive value; pN+, node-positive; QoL, quality of life.

**Table 7:** Risk of bias and applicability, per-patient diagnostic accuracy cohorts (QUADAS-2).

	Risk of Bias				Applicability		
Study	Patient Selection	Index Test	Reference Standard	Flow and Timing	Patient Selection	Index Test	Reference Standard
Kitagawa 2013 [13]	Low	Some concerns	Some concerns	Some concerns	Low	Low	Low
JCOG0302 2014 [26] frozen section	Low	Low	Low	Some concerns	Low	Some concerns	Low
*↳ JCOG0302* [26] *multiple paraffin sections*	Low	Some concerns	Some concerns	Some concerns	Low	Low	Low
Kinami 2016 [27]	Some concerns	Some concerns	Some concerns	Low	Low	Low	Low
Takahashi 2017 [21] infrared	Low	Some concerns	**High**	Low	Low	Low	**High**
*↳ Takahashi 2017* [21] *ordinary light*	Low	Some concerns	**High**	Low	Low	**High**	**High**
Shoji 2019 [28]	Some concerns	Some concerns	Some concerns	Some concerns	**High**	Some concerns	Some concerns
Mayanagi 2020 [9]	**High**	**High**	**High**	**High**	Low	Some concerns	**High**
SENORITA 2 2026 [22,30]	Some concerns	Some concerns	Low	Low	Some concerns	Low	Low
SENORITA phase III (Park 2024) [19,29]	Low	Some concerns	Low	Low	Low	Some concerns	Some concerns

**Legend**: Unit of analysis: patient. Domains and ratings follow QUADAS-2 [15], with signaling questions tailored to the review question (Section 2.7); a domain is rated low only where every signaling question is met. Italicized ↳ rows are the same cohort appraised under a second index test or protocol; the SENORITA phase III diagnostic cohort is appraised in the final row; its enhanced reference standard (cytokeratin immunohistochemistry with three step sections) underlies its applicability judgments, and a single limitation is not propagated across domains. Ratings are stated in each cell. Study-level rationale, including the reference-standard and partial-incorporation concerns, is given in Section 2.7 and Section 3.10.

## Data Availability

This review analyzed data extracted from published studies. The complete extracted 2 × 2 contingency data are provided within the article (Table 3 and the accompanying Table 4 block) and in the Supplementary Materials; all estimates are exact binomial (Clopper–Pearson) 95% confidence intervals computed in R (stats package) from those counts and are therefore reproducible directly from the published tables. The analysis scripts are available from the corresponding author upon reasonable request. The search strings, eligibility criteria and study selection process are reported in full in Section 2, Section 3.1, Figure 1 and Supplementary Table S1.

## Supplementary Materials

The following supporting information can be downloaded at https://www.mdpi.com/article/10.3390/cancers18203332/s1: Table S1: Search strategy, by database and register; Table S2: OSNA against paired histopathology, per lymph node; Table S3: Risk of bias and applicability, per-node molecular comparisons (QUADAS-2); Table S4: PRISMA 2020 checklist; Table S5: PRISMA-DTA checklist. Note S1: Extended legend to Figure 1, Study selection; Note S2: Extended legend to Figure 2, Per-patient detection rate and sensitivity.

## Abbreviations

The following abbreviations are used in this manuscript:

CI	Confidence interval
DFS	Disease-free survival
DSS	Disease-specific survival
ESD	Endoscopic submucosal dissection
FFPE	Formalin-fixed, paraffin-embedded
FNR	False-negative rate
HE	Hematoxylin and eosin
ICG	Indocyanine green
LSG	Laparoscopic standard gastrectomy
LSNNS	Laparoscopic sentinel node navigation surgery
NPV	Negative predictive value
OS	Overall survival
OSNA	One-step nucleic acid amplification
QoL	Quality of life
RI	Radioisotope
SNNS	Sentinel node navigation surgery
