# Supplementary material for: Sentinel Node Navigation Surgery in Gastric Cancer: A Systematic Review of Diagnostic Test Accuracy and Clinical Outcomes
Source: Cancers (Basel). 2026 Oct 15;18(20):3332. doi: 10.3390/cancers18203332 (PMC13702629; doi:10.3390/cancers18203332)
Supplement: Supplementary file 1 [file cancers-18-03332-s001.zip › cancers-4574689-supplementary.pdf]

Supplementary Materials

Sentinel Node Navigation Surgery in Gastric Cancer: A Systematic Review of Diagnostic Test Accuracy and Clinical Outcomes

Contents

Note S1. Extended legend to Figure 1. Study selection

Note S2. Extended legend to Figure 2. Per-patient detection rate and sensitivity

Table S1. Search strategy, by database and register

Table S2. OSNA against paired histopathology, per lymph node

Table S3. Risk of bias and applicability, per-node molecular comparisons (QUADAS-2)

Table S4. PRISMA 2020 checklist

Table S5. PRISMA-DTA checklist

Extended Figure Legends

Note S1. Extended legend: Figure 1. Study selection. Flow of records through the review, following the PRISMA 2020 template. The databases and registers searched, with dates last searched and records returned, are given in Table S1. Register records were screened alongside database records and none was removed as a duplicate; duplicate removal preceded screening and is shown in the identification tier. The single report identified by citation searching of reference lists is shown in the parallel right-hand column and entered the review at full-text assessment. Exclusion criteria were applied in a fixed order and screening stopped at the first criterion met, so each excluded report carries exactly one recorded reason; the criteria themselves are given in Section 2.2. Of the 19 reports retained at full text, 17 are reports of included studies and 2 are register records describing ongoing or unpublished trials, which are reported as ongoing research and contribute no data to the synthesis. Where several reports described the same cohort, all were retrieved and linked to that cohort, which was counted once; this is why the number of included reports exceeds the number of included studies. Thirteen reports contribute to the diagnostic accuracy stream and four to the outcome stream; the SENORITA phase III trial contributes to both and is counted once as a study.

Reports excluded at full-text review (non-English language)

Four full-text reports addressed the topic but were excluded under the English-language restriction (Section 2.2); each carries this single recorded reason. None is a primary diagnostic-accuracy cohort, so none would have contributed per-patient contingency data:

1. Miwa K. Endoscopic lymphatic mapping (ELM). Igaku no Ayumi 1994;170(11):940–941 (Japanese). Reason: non-English; methodological concept introduction, no per-patient accuracy data.

2. Kitagawa Y, Fujii H, Mukai M, et al. The validity of the sentinel node concept in gastrointestinal cancers. Nippon Geka Gakkai Zasshi (J Jpn Surg Soc) 2000;101(3):315–319 (Japanese). Reason: non-English; sentinel-node-concept validation paper.

3. Subgroup of Gastrointestinal Surgery, Surgical Society of the Chinese Medical Association; Oncology Surgical Group, Chinese Medical Doctor Association. Chinese expert consensus on function-preserving surgery for gastric cancer (2021 edition). Chin J Gastrointest Surg 2021;24(5):377–382 (Chinese). Reason: non-English; national consensus guideline.

4. Shin DJ, Son S-Y, Kim H-H. Sentinel node navigation surgery in gastric cancer. Korean J Clin Oncol 2014;10(1):6–11 (Korean). Reason: non-English; narrative review.

Note S2. Extended legend: Figure 2. Per-patient detection rate and sensitivity, with exact binomial 95% confidence intervals. Markers are point estimates and horizontal lines are Clopper–Pearson exact 95% confidence intervals, recalculated from the counts in each source report and printed beside each panel. Marker shape and fill denote the depth of the reference standard against which each estimate was measured. Marker size is uniform and encodes neither sample size nor study weight: no pooled estimate is computed, because each row pairs an index test with a reference standard of a different depth, so the estimates do not lie on a common scale and no row carries a weight (Section 2.8). The two panels use different horizontal scales and panel A is truncated at 50%, marked by the break on its axis; gridlines are drawn at the labelled ticks in both panels so that the difference in scale is apparent, and interval widths should not be compared by eye between panels. Indented italic rows are the same patient cohort reanalyzed under a second index test, reading basis or pathology protocol, and are not independent studies; JCOG0302 has one detection rate across both of its rows. Detection does not apply to the SENORITA phase III rows, whose analyzed cohort comprised patients in whom sentinel basin dissection had already succeeded. The dashed line at 90% in panel B is an orientation aid and not a tested threshold. Shoji 2019 accrued one node-positive patient, from which no sensitivity can be estimated.

Table S1. Search strategy, by database and register

Searches were run without language or date limits; the language restriction was applied at full-text screening. Strategies are reproduced as executed.

**Source**	**Platform / interface**	**Date last searched**	**Records returned**
PubMed/MEDLINE	PubMed	19 August 2026	603
Embase	Embase.com (Elsevier)	19 August 2026	965
Scopus	Elsevier	10 August 2026	852
Web of Science Core Collection	Clarivate	10 August 2026	660
CENTRAL	Cochrane Library (Wiley)	10 August 2026	62
ClinicalTrials.gov	clinicaltrials.gov	10 August 2026	30
UMIN-CTR	umin.ac.jp/ctr	10 August 2026	8
Citation searching	Reference lists of included reports and previous reviews	10 August 2026	1 report identified

Databases returned 3142 records and registers 38 (3180 in total). After removal of 1608 duplicates, 1572 records were screened.

PubMed/MEDLINE (PubMed interface)

("Stomach Neoplasms"[Mesh] OR "gastric cancer"[tiab] OR "gastric cancers"[tiab]
OR "gastric carcinoma*"[tiab] OR "gastric adenocarcinoma*"[tiab] OR "stomach cancer"[tiab]
OR "stomach carcinoma*"[tiab] OR "stomach neoplasm*"[tiab] OR "gastric neoplasm*"[tiab]
OR "gastric tumor*"[tiab] OR "gastric tumour*"[tiab] OR "early gastric cancer"[tiab] OR EGC[tiab])
AND
("Sentinel Lymph Node"[Mesh] OR "Sentinel Lymph Node Biopsy"[Mesh]
OR "sentinel node*"[tiab] OR "sentinel lymph node*"[tiab] OR "sentinel basin*"[tiab]
OR "sentinel navigation"[tiab] OR "navigation surgery"[tiab] OR "lymphatic basin*"[tiab]
OR "lymphatic mapping"[tiab] OR "basin dissection"[tiab] OR SNNS[tiab] OR SLNB[tiab]
OR SLNM[tiab])

Embase (Embase.com, Elsevier interface)

('stomach tumor'/exp OR 'stomach cancer'/exp
OR 'gastric cancer':ti,ab,kw OR 'gastric carcinoma':ti,ab,kw
OR 'gastric adenocarcinoma':ti,ab,kw OR 'stomach cancer':ti,ab,kw
OR 'stomach carcinoma':ti,ab,kw OR 'gastric neoplasm':ti,ab,kw
OR 'gastric tumor':ti,ab,kw OR 'gastric tumour':ti,ab,kw
OR 'early gastric cancer':ti,ab,kw)
AND
('sentinel lymph node'/exp OR 'sentinel lymph node biopsy'/exp
OR 'sentinel node':ti,ab,kw OR 'sentinel lymph node':ti,ab,kw
OR 'sentinel basin':ti,ab,kw OR 'sentinel navigation':ti,ab,kw
OR 'navigation surgery':ti,ab,kw OR 'lymphatic basin':ti,ab,kw
OR 'lymphatic mapping':ti,ab,kw OR 'basin dissection':ti,ab,kw
OR snns:ti,ab,kw OR slnb:ti,ab,kw)

Scopus (Elsevier interface)

TITLE-ABS-KEY("gastric cancer*" OR "gastric carcinoma*" OR "gastric adenocarcinoma*"
OR "stomach cancer*" OR "stomach carcinoma*" OR "stomach neoplasm*"
OR "gastric neoplasm*" OR "gastric tumor*" OR "gastric tumour*"
OR "early gastric cancer")
AND
TITLE-ABS-KEY("sentinel node*" OR "sentinel lymph node*" OR "sentinel basin*"
OR "sentinel navigation" OR "navigation surgery" OR "lymphatic basin*"
OR "lymphatic mapping" OR "basin dissection" OR "SNNS" OR "SLNB")

Web of Science Core Collection (Clarivate interface)

TS=("gastric cancer*" OR "gastric carcinoma*" OR "gastric adenocarcinoma*"
OR "stomach cancer*" OR "stomach carcinoma*" OR "stomach neoplasm*"
OR "gastric neoplasm*" OR "gastric tumor*" OR "gastric tumour*"
OR "early gastric cancer")
AND
TS=("sentinel node*" OR "sentinel lymph node*" OR "sentinel basin*"

OR "sentinel navigation" OR "navigation surgery" OR "lymphatic basin*"
OR "lymphatic mapping" OR "basin dissection" OR "SNNS" OR "SLNB")

CENTRAL (Cochrane Library, Wiley interface)

#1 MeSH descriptor: [Stomach Neoplasms] explode all trees
#2 (gastric NEAR/2 (cancer* OR carcinoma* OR adenocarcinoma* OR neoplasm* OR tumo*)):ti,ab,kw
#3 (stomach NEAR/2 (cancer* OR carcinoma* OR neoplasm*)):ti,ab,kw
#4 #1 OR #2 OR #3
#5 MeSH descriptor: [Sentinel Lymph Node] explode all trees
#6 MeSH descriptor: [Sentinel Lymph Node Biopsy] explode all trees
#7 (sentinel NEAR/2 (node* OR basin* OR navigation OR lymph*)):ti,ab,kw
#8 (lymphatic NEAR/2 (basin* OR mapping)):ti,ab,kw
#9 ("basin dissection" OR SNNS OR SLNB OR SLNM):ti,ab,kw
#10 #5 OR #6 OR #7 OR #8 OR #9
#11 #4 AND #10

ClinicalTrials.gov

Advanced search, all study statuses, no date or language limit.

Condition or disease:
gastric cancer OR stomach cancer

Other terms:
sentinel node OR sentinel lymph node OR sentinel basin
OR navigation surgery

UMIN-CTR

Free-text search, run separately in English and Japanese; no date limit.

English: gastric cancer + sentinel node
Japanese: 胃癌 + センチネルリンパ節

Table S2. OSNA against paired histopathology, per lymph node

Supplementary. Referenced from Section 3.8.

Study	Design	Comparison	Pts	Nodes	Sensitivity % (n/N) [95% CI]	Specificity % (n/N) [95% CI]	Concordance % (n/N) [95% CI]
**Yaguchi 2011 [23]**	• Assay validation<br>• Single center (NDMC)	• OSNA 250 copies/uL<br>• vs H&E	32	162	88.9 (40/45) [75.9–96.3]	96.6 (113/117) [91.5–99.1]	94.4 (153/162) [89.7–97.4]
**Kumagai 2014 [24]**	• Multicenter validation<br>• 4 Japanese hospitals	• OSNA vs histopathology<br>• All dissected nodes	61	394	83.3 (45/54) [70.7–92.1]	95.9 (326/340) [93.2–97.7]	94.2 (371/394) [91.4–96.3]
**Shimada 2020 [25] vs permanent histopathology**	• Prospective validation<br>• Single center (Keio)<br>• cT1N0M0	• OSNA vs permanent sections	43	439	63.6 (14/22) [40.7–82.8]	98.8 (412/417) [97.2–99.6]	97.0 (426/439) [95.0–98.4]
Shimada 2020 vs intraoperative histopathology	• Same cohort<br>• Second comparison	• OSNA vs intraoperative sections	43	214	80.0 (12/15) [51.9–95.7]	99.5 (198/199) [97.2–100.0]	98.1 (210/214) [95.3–99.5]

Legend

Unit of analysis: lymph node. The reference standard is the histopathology of the paired node half. These figures establish whether the assay and the pathologist agree about a single node, and not whether a tumor-free basin predicts a tumor-free field; they are not comparable with Table 3.

Exact binomial (Clopper–Pearson) 95% CI, recalculated from published counts.

Specificity is informative here, unlike Table 3, because paired-node histopathology can identify a false positive. False positives: Yaguchi 4; Kumagai 14; Shimada vs permanent 5; Shimada vs intraoperative 1.

Concordance is dominated by negative nodes and therefore overstates performance. An all-negative assay would score 72.2%, 86.3%, 95.0% and 93.0% respectively, against observed values of 94.4%, 94.2%, 97.0% and 98.1%.

Yaguchi and Kumagai were not early gastric cancer cohorts: 17/32 subserosal or serosa-exposed, and 29/61 T3 or T4, respectively. Shimada enrolled cT1N0M0 patients.

The two Shimada rows are the same 43 patients under different comparators and are not independent.

CI, confidence interval; H&E, hematoxylin and eosin; NDMC, National Defense Medical College; OSNA, one-step nucleic acid amplification.

Table S3. Risk of bias and applicability, per-node molecular comparisons (QUADAS-2)

Supplementary. Referenced from Section 3.10.

Study	Risk of bias Patient selection	Risk of bias Index test	Risk of bias Reference standard	Risk of bias Flow and timing	Applicability Patient selection	Applicability Index test	Applicability Reference standard
**Yaguchi 2011 [23]**	Some concerns	Low	Low	Low	**High**	Low	Low
**Kumagai 2014 [24]**	Some concerns	Low	Low	Low	**High**	Low	Low
**Shimada 2020 [25] vs permanent histopathology**	Low	Low	Low	Low	Low	Low	Low
Shimada 2020 vs intraoperative histopathology	Low	Low	Some concerns	Some concerns	Low	Low	Some concerns

Legend

Unit of analysis: lymph node. The index test is one-step nucleic acid amplification and the reference standard is the histopathology of the paired half of the same node. Because the two halves are examined by different methods, index test and reference standard are independent here, unlike the per-patient cohorts of Table 7, and a false positive can arise.

Cell shading is an aid to scanning only and carries no information beyond the printed rating: unshaded, low; light shading, some concerns; dark shading and bold, high. Ratings are the three-level graded judgments defined in Section 2.7 and are not the QUADAS-2 "unclear" category, which denotes insufficient reporting rather than a judged concern.

The two Shimada rows are the same 43 patients under different comparators and are not independent studies.

Row-specific notes. Yaguchi 2011 and Kumagai 2014 were not early gastric cancer cohorts (17 of 32 subserosal or serosa-exposed, and 29 of 61 T3 or T4, respectively); nodal deposits in advanced disease are larger than those in T1 tumors, so both are rated high for applicability of the included patients to the review question. Shimada 2020 enrolled cT1N0M0 patients and is the only comparison whose population matches the review question. Shimada 2020 against intraoperative histopathology is rated some concerns for the reference standard because intraoperative sections are a shallower comparator than permanent sections, and for flow and timing because the comparison covers 214 of the 439 nodes assessed against permanent histopathology.

These assessments bear on whether an assay and a pathologist agree about a single node. They do not bear on whether a tumor-free basin predicts a tumor-free field, and they are not comparable with Table 7.

Table S4. PRISMA 2020 checklist

Item numbers follow the PRISMA 2020 statement (Page et al., BMJ 2021;372:n71). The Topic column below carries short descriptors for orientation; the official PRISMA 2020 checklist, with the published item wording and the locations given here, is submitted as a separate file.

Section	Item	Topic	Location in manuscript
Title	1	Identify the report as a systematic review	Title
Abstract	2	Structured summary	Abstract
Introduction	3	Rationale	Section 1, paragraphs 1–4
	4	Objectives	Section 1, final paragraph
Methods	5	Eligibility criteria	Section 2.2
	6	Information sources	Section 2.3; Table S1
	7	Search strategy	Section 2.3; Table S1
	8	Selection process	Section 2.4; Figure 1
	9	Data collection process	Section 2.5
	10a	Data items: outcomes	Sections 2.5 and 2.6
	10b	Data items: other variables	Section 2.5; Table 2
	11	Study risk of bias assessment	Section 2.7
	12	Effect measures	Section 2.6
	13a	Synthesis: eligibility for each synthesis	Section 2.8
	13b	Synthesis: data preparation	Sections 2.5 and 2.6
	13c	Synthesis: tabulation and visual display	Tables 2–7; Figure 2
	13d	Synthesis: methods used	Section 2.8 (no meta-analysis performed)
	13e	Synthesis: heterogeneity exploration	Sections 2.8 and 3.7
	13f	Synthesis: sensitivity analyses	Not applicable; no pooled estimate
	14	Reporting bias assessment	Section 4.5, paragraph 2
	15	Certainty assessment	Section 2.7; Table 6

Section	Item	Topic	Location in manuscript
Results	16a	Study selection: flow	Figure 1; Section 3.1
	16b	Studies excluded at full text, with reasons	Figure 1
	17	Study characteristics	Section 3.2; Table 2
	18	Risk of bias in studies	Section 3.10; Table 7; Table S3
	19	Results of individual studies	Table 3; Table S2; Figure 2
	20a	Results of syntheses: characteristics	Section 3; Table 5
	20b	Results of syntheses: statistical results	Table 3; Figure 2
	20c	Results of syntheses: heterogeneity	Section 3.7
	20d	Results of syntheses: robustness	Not applicable; no pooled estimate
	21	Reporting biases	Section 4.5, paragraph 2
	22	Certainty of evidence	Table 6; Section 4.5, paragraph 3
Discussion	23a	Interpretation of results	Sections 4.3 and 5
	23b	Limitations of the evidence	Section 4.5, paragraphs 1 and 3
	23c	Limitations of the review process	Section 4.5, paragraph 2
	23d	Implications for practice and research	Sections 4.3 and 4.6
Other	24a	Registration and protocol	Section 2.1
	24b	Where the protocol can be accessed	Section 2.1
	24c	Amendments to registered information	Section 2.1 (decision not to pool taken at synthesis)
	25	Support and funding	Funding statement
	26	Competing interests	Conflicts of Interest statement
	27	Availability of data, code and materials	Data Availability Statement

Table S5. PRISMA-DTA checklist

PRISMA-DTA (McInnes et al., JAMA 2018;319:388–396) follows the PRISMA item structure and adds items specific to diagnostic test accuracy. The official PRISMA-DTA checklist, with the published item wording and the locations given below, is submitted as a separate file.

**DTA-specific topic**	**Location in manuscript**
Target condition being diagnosed	Section 2.2 (regional lymph node metastasis)
Index test, including its definition and threshold	Section 2.2; Table 2
Reference standard, including its definition	Section 2.2; Table 2; Section 2.7 (tailoring decision 2)
Rationale for the index test and its intended clinical role	Section 1, paragraphs 3–4
Unit of analysis (patient or node)	Section 2.6; legends to Table 3 and Table S2
Definition and handling of indeterminate or missing index test results	Section 2.6; Table 3 legend
Reconstruction of 2 × 2 contingency counts	Section 2.5; Table 3 footnotes a-c
Methods of estimating accuracy and confidence intervals	Section 2.6
Rationale for not pooling estimates	Sections 2.8 and 3.7; Figure 2 legend
Risk of bias assessed with QUADAS-2, with tailored signaling questions	Section 2.7; Table 7 legend
Individual study results as 2x2 counts with estimates and intervals	Table 3; Table S2; Figure 2
Time interval and any intervention between index test and reference standard	Table 2; Table 7 (flow and timing)
Distribution of severity of the target condition	Section 3.7 (macrometastasis vs micrometastasis); Table 2
Applicability of the findings to the review question	Table 7; Table S3; Section 4.5, paragraph 1

Note. Specificity and overall accuracy are not reported for the per-patient analyses because full regional lymphadenectomy served as the reference standard, so a false positive cannot arise (Section 2.6). JCOG0302 is the single exception and is reported in Table 3.
